# Supplementary figures and images for: Recent transposable element bursts are associated with the proximity to genes in a fungal plant pathogen
Source: PLoS Pathog. 2023 Feb 14;19(2):e1011130. doi: 10.1371/journal.ppat.1011130 (PMC9970103; doi:10.1371/journal.ppat.1011130)

**A**

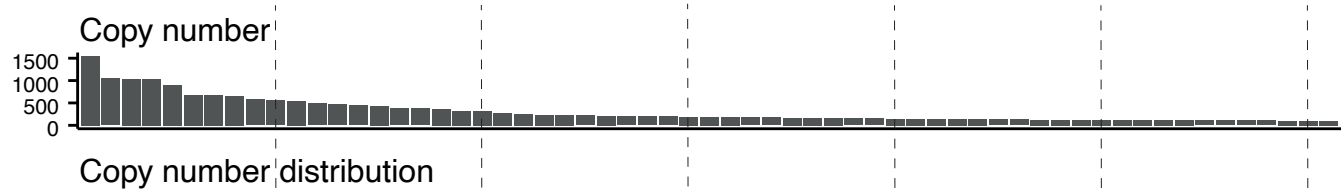

**B**

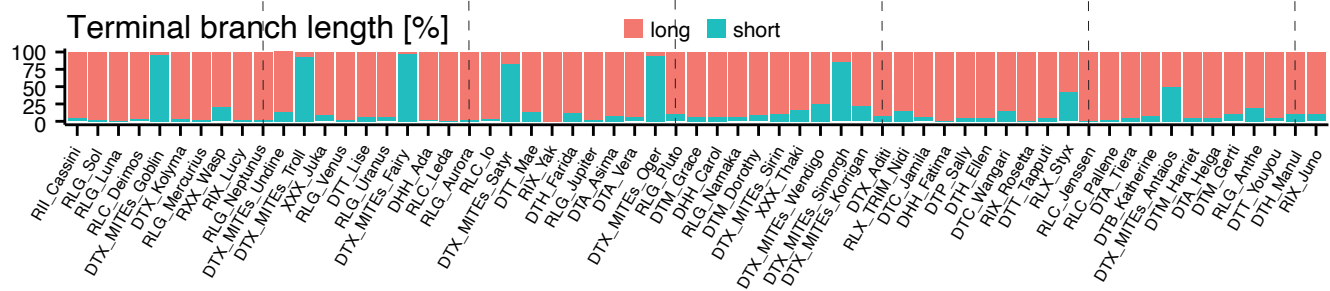

Supplement: S2 Fig — (A) Total copy numbers. (B) Long (> 0.00001; red) and short (≤0.00001; blue) terminal branch lengths of individual copies characterizing two classes of divergence times. (PDF) [file ppat.1011130.s002.pdf]

**A**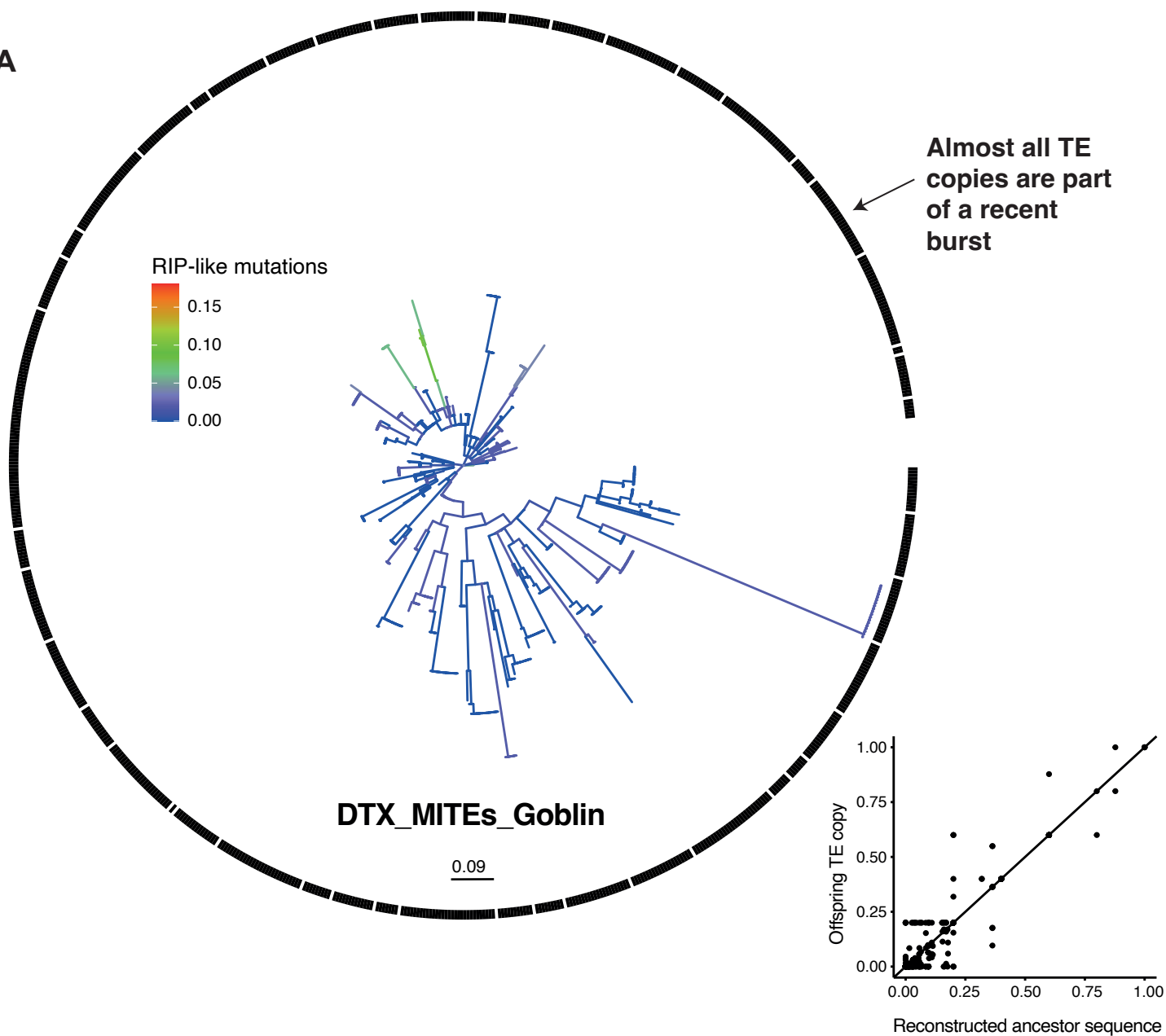**B**

GC niche [%]

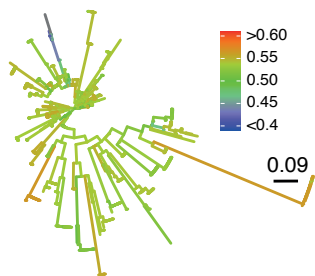**C**

Large RIP affected region [%]

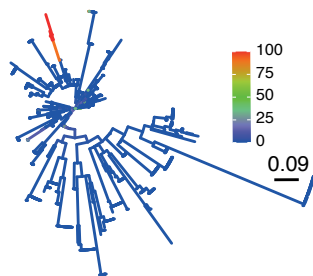**D**

GC fragment [%]

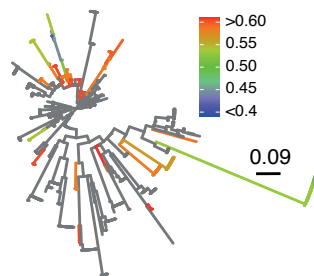**E**

Closest gene [bp]

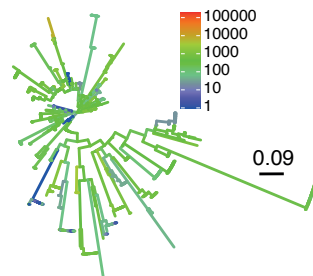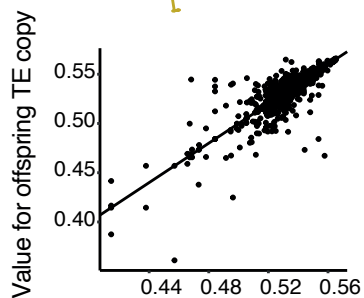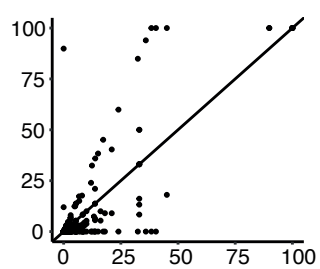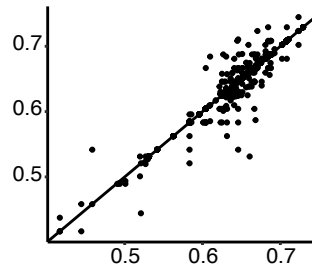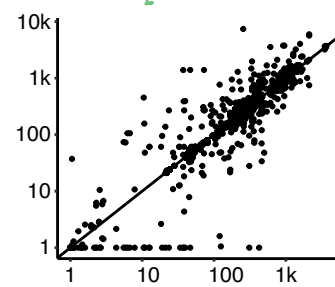

Supplement: S3 Fig — The black bar marks the different burst clades. The dot plot shows the changes in RIP-like mutations from the ancestor to offspring for all internal and terminal branches from the ancestral state reconstruction. (B-E) Phylogenetic trees and ancestor-offspring changes for (B) the GC content of the niche, (C) the overlap of the niche with large RIP affected regions, (D) the GC content of the copy and (E) the distance to the closest gene. (PDF) [file ppat.1011130.s003.pdf]

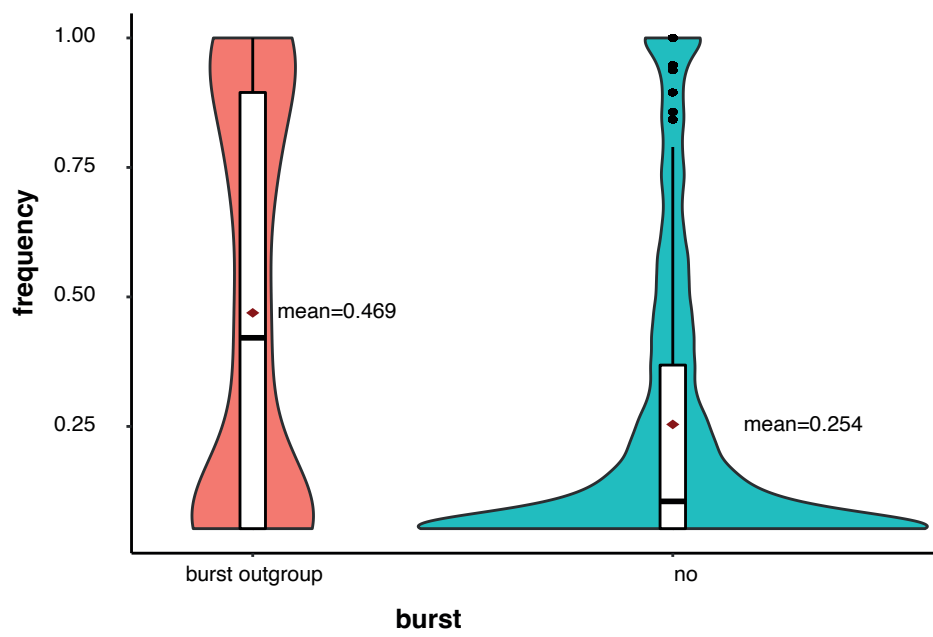

Supplement: S4 Fig — Comparison of TE copy frequency between outgroups of burst and all other TE copies. (PDF) [file ppat.1011130.s004.pdf]

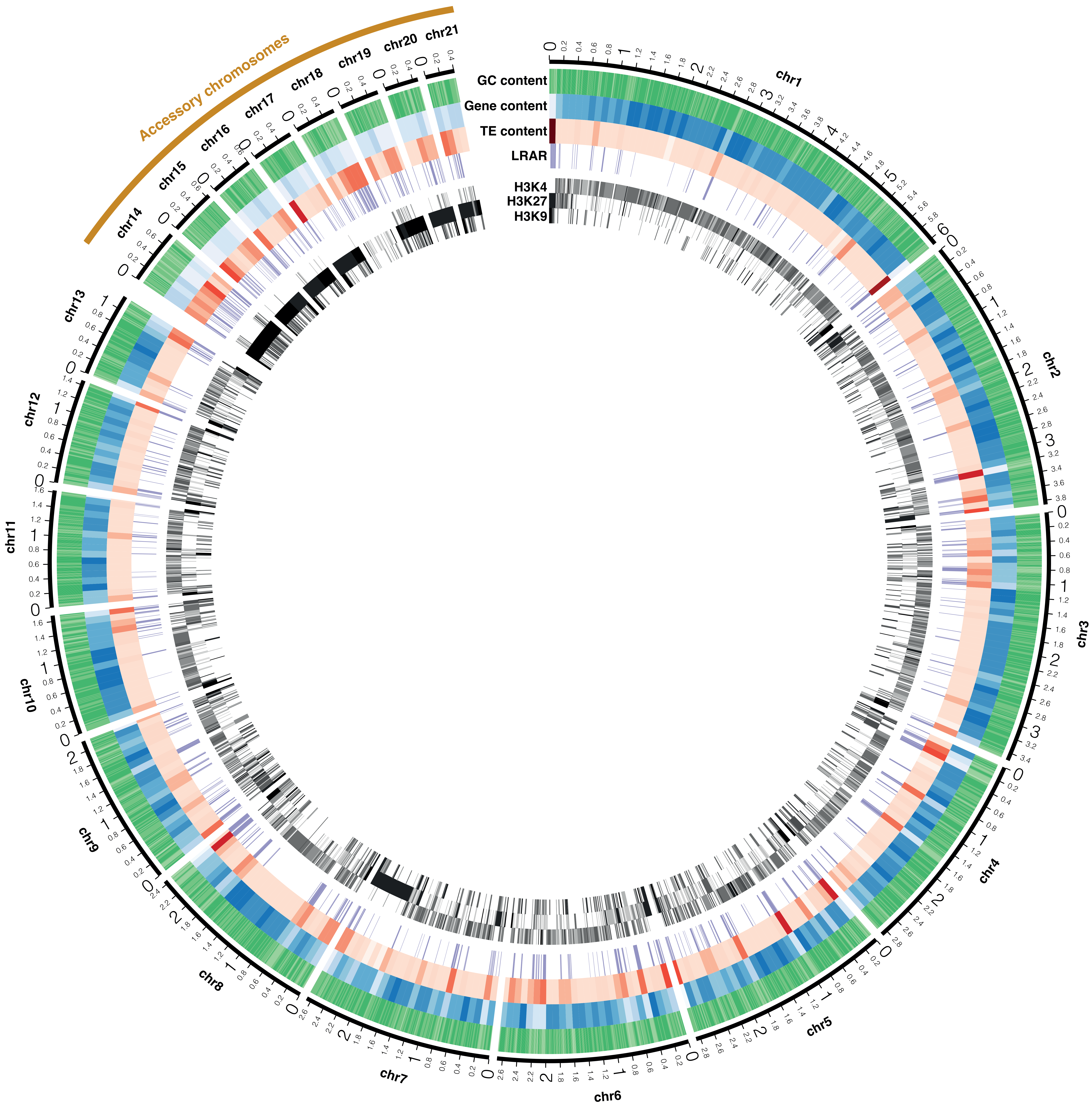

Supplement: S5 Fig — Circos plot showing the genomic environment of the reference genome IPO323 (Dutch isolate). Description from outside to inside contains the GC content, gene content and TE content in windows of 10kb, the presence of large RIP affected regions (LRAR), and the indication of the histone marks H3K4, H3K27 and H3K9. Chromosomes 1–13 are core chromosomes that are present in each isolate, while chromosomes 14–21 are accessory chromosomes, that are not shared among all isolates. (PDF) [file ppat.1011130.s005.pdf]
