# Supplementary material for: Recent transposable element bursts are associated with the proximity to genes in a fungal plant pathogen
Source: PLoS Pathog. 2023 Feb 14;19(2):e1011130. doi: 10.1371/journal.ppat.1011130 (PMC9970103; doi:10.1371/journal.ppat.1011130)
Supplement: S6 Fig — Due to the high number of nested insertions and partially deleted fragments, we aligned only coding regions. (PDF) [file ppat.1011130.s006.pdf]

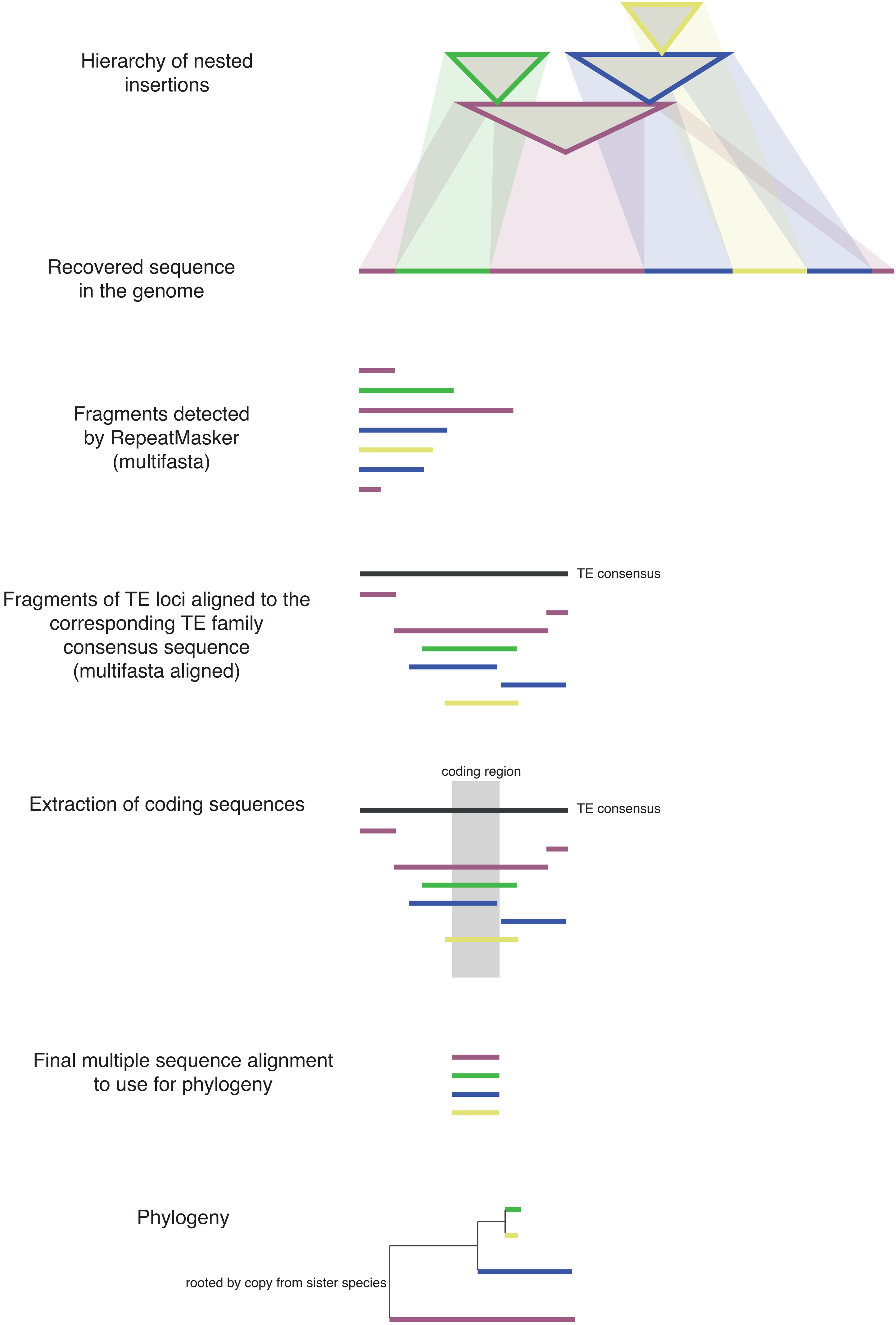
Hierarchy of nested insertions
Recovered sequence in the genome
Fragments detected by RepeatMasker (multifasta)
Fragments of TE loci aligned to the corresponding TE family consensus sequence (multifasta aligned)
TE consensus
Extraction of coding sequences
coding region
TE consensus
Final multiple sequence alignment to use for phylogeny
Phylogeny
rooted by copy from sister species
