# Supplementary material for: Recent transposable element bursts are associated with the proximity to genes in a fungal plant pathogen
Source: PLoS Pathog. 2023 Feb 14;19(2):e1011130. doi: 10.1371/journal.ppat.1011130 (PMC9970103; doi:10.1371/journal.ppat.1011130)
Supplement: S1 Fig — The Z. tritici specific family names are according to Badet et al (2020) [52]. (PDF) [file ppat.1011130.s001.pdf]

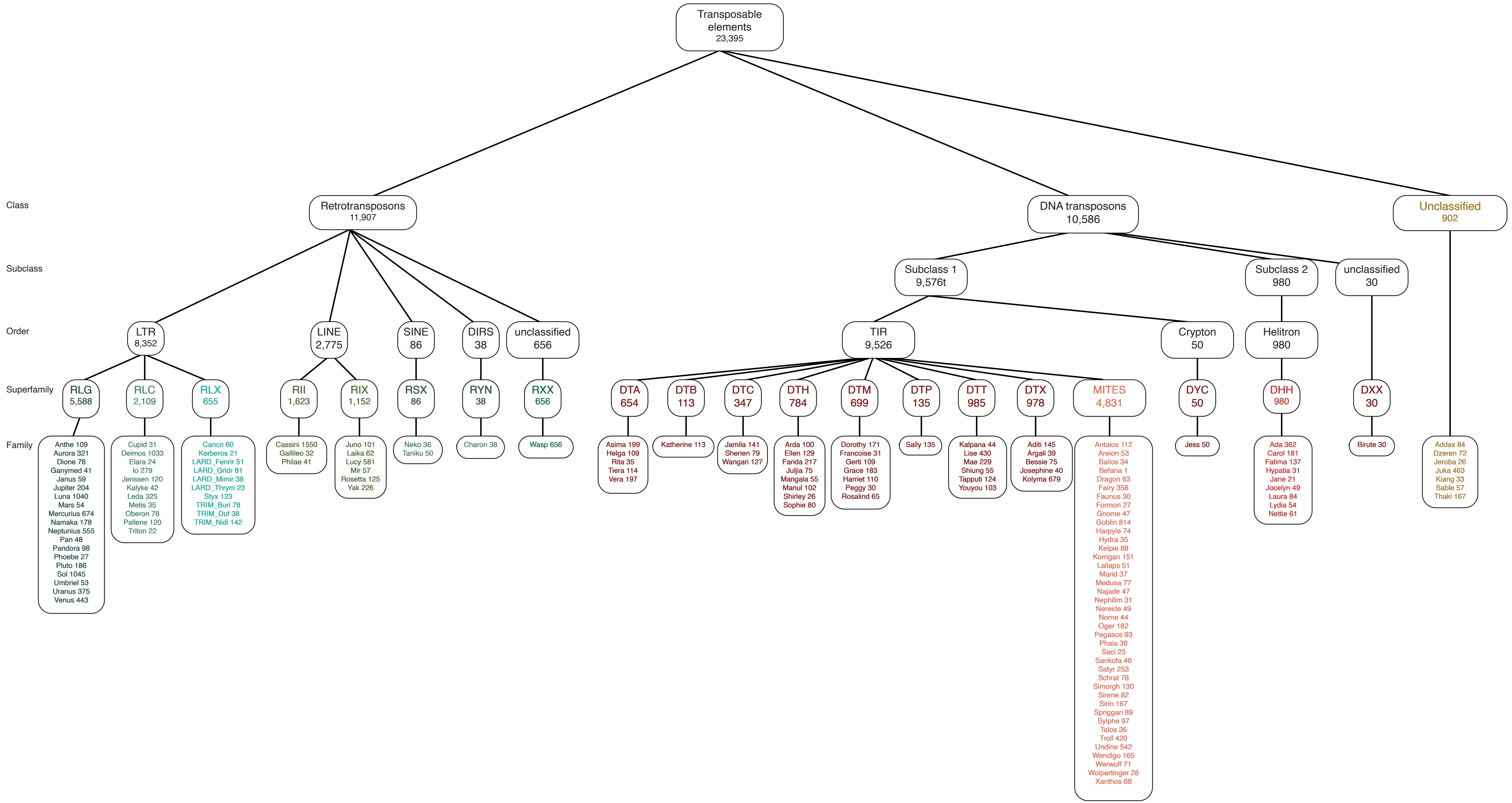

Transposable elements
23,395
Class
Subclass
Order
Superfamily
Family
Retrotransposons
11,907
DNA transposons
10,586
Unclassified
902
Subclass 1
9,576t
Subclass 2
980
unclassified
30
LTR
8,352
LINE
2,775
SINE
86
DIRS
38
unclassified
656
TIR
9,526
Crypton
50
Helitron
980
RLG
5,588
RLC
2,109
RLX
655
RII
1,623
RIX
1,152
RSX
86
RYN
38
RXX
656
DTA
654
DTB
113
DTC
347
DTH
784
DTM
699
DTP
135
DTT
985
DTX
978
MITES
4,831
DYC
50
DHH
980
DXX
30
Anthe 109
Aurora 321
Dione 78
Ganymed 41
Janus 59
Jupiter 204
Luna 1040
Mars 54
Mercurius 674
Namaka 178
Neptunius 555
Pan 48
Pandora 98
Phoebe 27
Pluto 186
Sol 1045
Umbriel 53
Uranus 375
Venus 443
Cupid 31
Deimos 1033
Elara 24
Io 279
Jenssen 120
Kalyke 42
Leda 325
Metis 35
Oberon 78
Pallene 120
Triton 22
Cancri 60
Kerberos 21
LARD_Fenrir 51
LARD_Gridr 81
LARD_Mimir 38
LARD_Thrym 23
Styx 123
TRIM_Buri 78
TRIM_Duf 38
TRIM_Nidi 142
Cassini 1550
Gallileo 32
Philae 41
Juno 101
Laika 62
Lucy 581
Mir 57
Rosetta 125
Yak 226
Neko 36
Taniku 50
Charon 38
Wasp 656
Asima 199
Helga 109
Rita 35
Tiera 114
Vera 197
Katherine 113
Jamila 141
Sherien 79
Wangari 127
Arda 100
Ellen 129
Farida 217
Juljia 75
Mangala 55
Manul 102
Shirley 26
Sophie 80
Dorothy 171
Francoise 31
Gerti 109
Grace 183
Harriet 110
Peggy 30
Rosalind 65
Sally 135
Kalpana 44
Lise 430
Mae 229
Shiung 55
Tapputi 124
Youyou 103
Aditi 145
Argali 39
Bessie 75
Josephine 40
Kolyma 679
Antaios 112
Areion 53
Balios 34
Befana 1
Dragon 63
Fairy 358
Faunus 30
Formori 27
Gnome 47
Goblin 814
Harpyie 74
Hydra 35
Kelpie 88
Korrigan 151
Lailaps 51
Marid 37
Medusa 77
Najade 47
Nephilim 31
Nereide 49
Norne 44
Oger 182
Pegasos 93
Phaia 38
Saci 23
Sankofa 46
Satyr 253
Schrat 78
Simorgh 130
Sirene 82
Sirin 167
Spriggan 89
Sylphe 97
Talos 36
Troll 420
Undine 542
Wendigo 165
Werwolf 71
Wolpertinger 28
Xanthos 68
Jess 50
Ada 362
Carol 181
Fatima 137
Hypatia 31
Jane 21
Jocelyn 49
Laura 84
Lydia 54
Nettie 61
Birute 30
Addax 84
Dzeren 72
Jeroba 26
Juka 463
Kiang 33
Sable 57
Thaki 167
