# Supplementary material for: Recent transposable element bursts are associated with the proximity to genes in a fungal plant pathogen
Source: PLoS Pathog. 2023 Feb 14;19(2):e1011130. doi: 10.1371/journal.ppat.1011130 (PMC9970103; doi:10.1371/journal.ppat.1011130)
Supplement: Attachment — Submitted filename: Response_reviewers.docx [file ppat.1011130.s009.docx]

Dear Dr. Thomma,

We are very grateful for the thorough review process and the numerous pertinent requests to revise our manuscript. We have now addressed all raised issues and have very substantially revised our submission including nearly all figures.

With best regards,

Daniel Croll

########################################

*Reviewer #1: In many plant pathogenic fungi as well as other organisms, transposable elements (TEs) exist abundant in genomes and contribute to their evolution. In these fungi, however, little is known about how bursts of TE copies have occurred, due to the insufficiency of high-quality genome assemblies. This paper characterized TE burst events of a wheat pathogen Zymoseptoria tritici using complete (telomere-to-telomere) genome sequences of 19 isolates. By a phylogenetic approach, the authors divided TEs with higher copy numbers into the copies derived from burst events and the others. The analysis revealed that certain TE families experienced the bursts one or several times and that TEs in the burst clade tended to be located closer to genes. This paper showed the association between the TE bursts and the genomic environments at each TE family level. This paper along with the other accumulated evolutionary papers of this fungus can contribute to the understanding of how the pathogen evolution has been driven, which can give a certain impact on related research fields of other plant pathogens. However, there are fatal data labeling errors and untransparent descriptions, which make it harder to judge whether some of the authors’ interpretations are correct or not, as listed below. These data should be reanalyzed and reinterpreted carefully. Even though the mistakes, the overall approaches toward the scientific question seem reasonable so the manuscript will be fine as long as the errored data and accompanied interpretations will be corrected without any contradiction.*

**RESPONSE** : We are very grateful for pointing out the errors contained in the figures. We have carefully re-checked the data to ensure that all is accurate. Where relevant, we have provided further explanations if the presented data was without any errors but lacked context.

*Reviewer #2: I think that the paper summarize a good work.*

*The authors uses 19 isolates to describe the history of TE evolution in different Z. tritici. They characterize all TEs in all 19 isolates, they find specific characteristics of each TE family and using two of them, they try to describe burst of expansion. It is known that genome evolution is shaped by TE burst and therefore, they try to connect TE burst to evolution of individual strains isolated in different part of the world.*

*although the paper is readable, in some parts there are many assumptions and in the last paragraph of the results, there is little indication where to find the interesting results in the figures. Ultimately, the paper results difficult to read for people that are not in the field of TE evolution*

**RESPONSE** : We regret the lack in clarity and have improved all sections of the manuscript to provide a clearer picture of our findings.

*Reviewer #3: The manuscript “Recent transposable element bursts triggered by insertions near genes in a fungal plant pathogen” by Oggenfuss and Croll is a fascinating deep dive into the transposable element dynamics of the fungal pathogen Zymoseptoria tritici. Taking advantage of a recent population genomic dataset of 19 strains, the authors take a unique approach of evaluating the genomic niche that different TEs occupy within the genomes. They determine what properties of these niches correspond with given TE superfamilies and uncover interesting patterns with regard to which elements are currently undergoing expansions within the population. The work is very methodical and encompassing, however I feel that a lot of improvement should be made before a final version is ready for a broader scientific audience. In particular, I believe that the current draft will be very difficult to understand for readers that are not experts on transposable elements. I hope that the following comments will help the authors rework their manuscript.*

**RESPONSE** : We have carefully revised the manuscript in particular in regards to our framing and wording in order to make the text more accessible.

*Reviewer #4: I have now read and considered the manuscript entitled Recent transposable element bursts triggered near genes in a fungal plant pathogen*

*Overall this is a well written manuscript that leverages 19-complete telomere to telomere assemblies to examine the evolution of transposon families that have undergone recent ‘bursts’ within Zymoseptoria tritici. The authors have uncovered some interesting patterns, for example that small non-autonomous transposons (mites) are more likely to escape genome defence mechanisms and reside near genes. The authors also explore in more detail two very large transposons that have undergone recent expansion (Cassini, Deimos). I do not find any flaws in the analyses or data presented, however I found the amount of information presented in the figures overwhelming, which made it difficult to distil out the main messages that the authors wish to communicate. I realise that this is likely a shortened revision of a previous manuscript based on my observation that the text references 5 figures but there are seven figures in the material. Also, there are more panels listed in the figure legends than there are in the figures. So I suspect that I am asking the authors to even further simplify a longer version of a prior draft manuscript.*

**RESPONSE** : Thank you very much for the careful reading. We have indeed heavily revised the manuscript in multiple round but this was done internally and not following peer-review. We double-checked that all figure elements and supplementary information were cited.

*Below are some comments/suggestions that I hope will improve clarity and help the authors better communicate their main findings.*

**RESPONSE** : We can confirm that we have reduced the information displayed in the main figures and fixed any errors in figure legends. For example, we condensed the information in figure 1 and split figure 2. We modified panels in figure 4. We apologize for this and hope that the revised version provides now the expected clarity.

***Part II – Major Issues****: Key Experiments Required for Acceptance*

*Please use this section to detail the key new experiments or modifications of existing experiments that should be absolutely required to validate study conclusions.*

*Generally, there should be no more than 3 such required experiments or major modifications for a "Major Revision" recommendation. If more than 3 experiments are necessary to validate the study conclusions, then you are encouraged to recommend "Reject".*

*Reviewer #1: 1. There are several fatal errors in Figure 3.*

*1.1. Some TE families seem mislabeled in Figure 3A-E, which leads to the contradiction with Figure 3F and texts. For example, the description in lines 177-178 contradicts Figure 3B. The median for DTX_MITE_Goblin and RLC_Deimos (the fourth or the fifth from the left) seems below 40% or over 60%, respectively, in the figure, against the text description. Moreover, the values shown in Figure 3B-E do not correspond to that shown in Figure 3F. It can be easily understood from the plots of DTX_MITE_Goblin [the highest RIP-like mutation in (E)], RLC_Deimos [shorter length in (C)], and RLG_Uranus [the highest nucleotide diversity in (D)], for instance.*

**RESPONSE**: This is correct - there was a shift in the x-axis labeling, which induced a bias to the results in Figure 3. We added the correct x-axis labeling and additionally added a legend for the superfamily legends.

*1.2. It seems strange that the scale for “RIP-like mutation” in Figure 3E and F is inconsistent, without any explanation.*

**RESPONSE**: We adapted the legend in Figure 3F to the correct dimensions and added more information as well.

*1.3. The explanation for each panel in the legend is off by one or two, probably because two panels [former (A) and (G)] were moved to Supplementary Figure S2A. Please correct the alphabet number in the Figure 3 legend.*

**RESPONSE**: we thank the reviewer for detecting this mistake, and we changed the legend accordingly.

*There are additional suggestions for Figure 3 as follows.*

*1.4. Please add the explanation to the legend for what the red horizontal line in Figure 3B and C indicates. In line 176, please describe what percentage is “the genome-wide GC content”. Does the red horizontal line in Figure 3B indicate that?*

**RESPONSE**: we added the missing information both in the legend and in the main text

*1.5. In Figure 3D, can the nucleotide diversity be shown by a logarithmic scale? Otherwise, it is hard to understand that “MITEs tend to have higher nucleotide diversity” as described in line 184.*

**RESPONSE**: we changed Figure 3D to a logarithmic scale and additionally added the color of the superfamily.

*1.6. If the box plots and/or bar graphs in Figure 3A-E will be colored distinguishable for each superfamily, it will be easier to catch some trends discussed.*

**RESPONSE**: the coloring was already according to superfamily, we added the legend to the figure as well

*2. There are also a suggestion and errors in Figure 4.*

*2.1. Why DTX_MITE_Goblin was excluded from the analysis in Figure 4A?*

**RESPONSE**: we added DTX_MITE_Goblin to Figure 4A. This family has no apparent burst over time and the line remains flat.

*2.2. In Figure 4D, what the colors indicate is inconsistent between the illustrated or text legends. The red and blue colors respectively show “no burst” or “burst” on the figure, but opposite in the text legend in lines 760-761.*

**RESPONSE**: we changed the legend of Figure 4D and changed the legend text in the Figure 5 so that it is corresponding to Figure 5 now

*2.3. The discussion in line 261 is inconsistent with the result in Figure 4D. The “lower” would be “higher”.*

**RESPONSE**: Thank you. We adjusted the text

*3. Some results are described without showing data, exemplified as follows. It is desirable to visualize or summarize the data in additional figures or tables.*

*3.1. The description in line 155, “TE insertions are at higher density on accessory chromosomes (Figure 2B)”, is not supported by Figure 2B which shows “number of copies” but not “density”.*

**RESPONSE**: we agree with the reviewer. Wwe added the corresponding density plots to Figure 2B

*3.2. As for the description in lines 217-218 (“Overall, around 50% ... several bursts”), could it be shown which TE families experienced how many times bursts in how many genomes out of 19?*

**RESPONSE**: Evidence for a particular TE burst is essentially never stemming from a single genome alone. Burst reconstructions include copies from multiple or sometimes all genomes. Hence, we think that even though some bursts are “recent”, they still are not based restricted to an individual genome. We added a bit more text to make this feature of *Z. tritici* bursts more clear.

*3.3. Could the data for a discussion in lines 322-324 (“We found ... most analyzed genomes).”) be shown?*

**RESPONSE**: We added the corresponding Figure as new Supplementary Figure S4

*4. Please add a clear explanation for the difference between “RIP index” and “RIP-like mutations” analyzed in Figures 2F, 4B and D.*

**RESPONSE**: we removed the results related to the RIP-index, as we did not refer to the RIP-index any further.

*5. The trend that burst TE copies tended to be located near genes (Figure 4D) is not observed in some TE families (e.g. RII_Cassini and DTX_MITEs_Goblin) (Figure 5). Please add the discussion about that.*

**RESPONSE**: We adapted the text in the results and discussion.

*6. It seems better to change the paper title from “… triggered by …” to “… associated with …” or other such milder expression, since this paper shows no direct evidence for the causal relationship expressed by the current title. Lines 130-131 (“The comprehensive … number expansions.”) and 263-264 (“Our analyses ... coding sequences.”) also seem to be over-discussed.*

**RESPONSE**: We agree that we are not showing the association at a direct molecular level but rather using a statistical approach. We changed the writing in the mentioned section. We have also revised the title accordingly.

*Reviewer #2:*

*Line 140: positive correlation needs to be assessed with statistical means.*

**RESPONSE**: We added the statistics from the original literature

*Line 147: what do you mean with the sentence “Only few TE insertions are fixed among genomes (n = 122; Figure 1D) and consist predominantly of MITEs”. Reading the paragraph, I understand that it refers to a specific family present in multiple isolate or only in one but reading the figure legend is more about location rather than presence/absence of a family:” Allele frequencies of TEs at orthologous insertion loci among genomes”. Can you explain better in the text?*

**RESPONSE**: We agree that a clarification is needed here. In fact, Figure 1D is showing the locus-specific frequency of insertions, with “fixation” meaning that a copy of the same TE family is present in this locus in all 19 genomes. As fixation or high frequencies in a locus are rare, we meant to show that fixed TEs do not have a strong impact, and that we do not need to remove orthologous copies. Having many fixed TEs would mean we would need to add additional tests, to be sure that these are in fact orthologous TEs and not multiple individual insertions. We clarified this in the text and added some details to the methods.

*Line 150: I understand what the author did but I think that it should be explained better. I can understand that the author think that all readers need to know which marker are associated with euchromatin and which with heterochromatin but at the moment I think that this is not a common knowledge therefore I think that you need to explain which marker goes with either heterochromatin or euchromatin*

**RESPONSE**: We agree that more information is needed. We added the markers to the text, added the results for facultative heterochromatin, and added more information on chromatin marks in the introduction.

*Line 155: “, but TE insertions are at higher density on accessory chromosomes”. Honestly, I can not see where this is reported in fig 2B. In contrast I see that that bar in figure 2B regarding the panel chromosome has a higher number of TE in the core genome. I think that the authors should find a different way to plot the density in function of core/accessory chromosome size*

**RESPONSE**: We added plots showing the TE density (number of TEs per bp) to allow a proper comparison.

*Line 184: “MITEs tend to have higher nucleotide diversity at 185 similar copy numbers compared to other TE families (Figure 3D, F)”. I find difficult to see this. In figure 3D there are many MITs elements but only one has an high Nucleotide diversity: DTX_MITE_Toll. The rest is normal.*

**RESPONSE**: There was a shift in the y-axis labeling that is now corrected. This clarifies the pattern that MITEs have a tendency of higher nucleotide diversity. As mentioned by reviewer #1, we also changed Figure 3D to log-scale to make the differences more appreciable.

*Line 185: looking at RIP, on MITE_Goblin has an high value of RIP compared to others.*

**RESPONSE**: This is the same issue with the shift in y-axis labeling. With the correct naming, it is now apparent that RIP is low in DTX_MITEs_Goblin, and the high RIP values stem from RLC_Deimos. We apologize for this issue.

*Line 193: “Our findings show that recent bursts are characterized by high copy numbers, but genomic defences and the length of TEs create complex outcomes for individual TE families.” Honestly, I do not understand where this come from and what this means*

**RESPONSE**: We removed the sentence as this did not convey a useful summary of the paragraph. The rephrased paragraph should hopefully be clearer.

*The paragraph entitled “NICHE CHARACTERISTICS OF TRANSPOSABLE ELEMENT ACTIVATION” is very difficult to read. The paragraph is short and refers to fig 5 and 6 where for each figure A-E panels. The authors describe their findings but do not refer in an appropriate way where to find the information that support their statements. This paragraph should be written better and explained better the results*

**RESPONSE**: We re-wrote this paragraph and rearranged the figure. We hope that this makes this section now more readable.

*Reviewer #3: I have only one major concern with the analyses, which involves RIP. Given that RIP is a stochastic process, some of the underlying assumptions that the authors appear to have made may be violated. Firstly, RIP becomes inefficient for repeat lengths below ~ 500 bp (see https://doi.org/10.1038/ncomms4509), thus the smaller TEs like MITES are likely undergoing fundamentally different evolutionary processes.*

**RESPONSE**: We agree with these important boundaries to RIP as we understand it currently. Our writing did not stress these facts well enough. We extended now the relevant section of the introduction and reformulated the discussion where we touched on RIP.

*This could cause a number of issues when lumping all of the analyses together. For example, using the same branch length cut offs for both the MITEs and LTRs could be underestimating the number and nature of burst events.*

**RESPONSE**: We agree that RIP will very likely have unequal effects on different categories of TEs. For this reason, we analyzed and discussed groups of TEs (MITEs, *Copia*+mdg4, LINE and others), separately. We did indeed use a common cut-off to collapse branches as specified in the methods that was very small and that was largely insensitive. However, given the complexity of reasons how RIP may have affected different TEs (length, location, geographic distribution, etc.), we feel that there would not be a robust way to adjust the cut-off per TE. Hence, we believe that discussing TEs as major groups is more cautious. We changed the text in the methods and results to make this more apparent.

*Second, it seems that the authors have come to some concrete conclusions about precisely which element is the ‘master’ copy of burst events. It is not clear from the current draft exactly how this was done, but both the nature of RIP, and the fact that the MITEs are non-autonomous, should make this very difficult if not impossible.*

**RESPONSE**: We agree that this was not clearly described. We also want to emphasize again that it is nearly impossible to define the precise copy at the origin of a burst given the divergence since the burst and the possible lack of a reference genome reproducing the exact copy (despite using 19 such genomes of the same species). Rather, we wanted to describe the characteristics of element(s) that are credibly very close to the initiation of the burst. We added missing information to the methods and results.

*Lastly, as the authors bring up in the discussion, some strains have lost RIP or components of it, which would mean that any TE dynamics in those strains would be fundamentally different from the others. It is not mentioned which strains have lost it, and as the results never break down the dynamics by strain, one cannot currently evaluate this. Some of these points could directly impact the main conclusion, that bursts are driven by insertions near genes. For example, is the reported copia element burst in a strain without RIP? Is it then true that bursts originate from gene rich regions, or does that strain have a unique distribution of TEs because it has lost its genome defense machinery? Likewise, although the MITEs are in gene rich regions, where are the protein coding elements? Isn’t that the more important question with regard to what’s driving the expansions?*

**RESPONSE**: We added the missing information on the RIP mechanism in *Z. tritici* and the relevant literature reporting the geographic scope of RIP (e.g. Feurtey et al, 2022) to the introduction and discussion. Even though there is evidence that RIP might be reduced or even lost within the species, there is significant recent gene flow among continents. Hence, each analyzed genotype will be admixed to some degree from different geographic origins. Hence, RIP could also vary within the genome due to admixture instead of the efficacy of RIP alone. We also added to the discussion that the two "isolate-specific" bursts in RII_Cassini might have happened due to a reduced efficiency of RIP. These are the closest examples suggesting a geographic link that we can report using the 19-genome panel alone.

*In addition to this issue, the introduction could be improved. Large parts currently read as a list of interesting facts that are only loosely tied together, and are sometimes repetitive. e.g. Line 54 talks about silencing of TEs, and the same information is presented again on Line 120. Other important information is either hidden in the supplement (like what the codes RLC, RLG etc. mean, or even definitions for Copia and Gypsy), or not presented until the discussion, such as much of the information about RIP. Furthermore, the start of the results section seems to confound analyses from the previous study (Badet et al., 2020) with what is done here. e.g. Line 140: "show a positive correlation", this does not appear evident from Figure 1, but is clear from Badet et al., 2020 figure 5A. The authors should be clear about what information has been presented again for the sake of this study and what are new analyses.*

**RESPONSE**: We changed the results to be more concise and clear about the starting point of our investigations. We also added information concerning the naming of TEs in the methods.

*Reviewer #4: The authors should include a better introduction to transposon families, in particular the three families that they focus on throughout the results (MITEs, Copia, Gypsy).*

**RESPONSE**: We added the missing information to the introduction and methods (where relevant for describing groups of TEs)

*Introduction: For non-specialist readers it may be helpful to briefly introduce Class I vs. Class II TEs. Many of the TE names listed in the Manuscript use the Wicker classification abbreviation (RLX, RLC,RLG) but this is never defined. May be enough to define Copia/Gypsy, LINEs and MITEs? I also note a new name for the TE family Gypsy has been proposed, to avoid some of the negative history associated with this name, I wonder if the authors would consider adopting this new name. Lines 96-101 may be a good place to revise and consider adding this information in, the current text here describes the loss of Long-terminal repeats will probably not make much sense to someone who is not familiar with TE families that contain these features.*

**RESPONSE**: We changed the introduction to give a better overview on TEs. We are aware of the proposition to change the offensive “Gypsy” name, and changed the occurrences of this old name in the figures and added the Wei et al, 2022 manuscript. Yet, we are not aware that the TE community already agreed on a new name. Using the three-letter code from Wicker et al, 2007 was our way to go around this problem, until it is settled.

*The loss of dim2 in some populations of Z. tritici is an important point that is not very clearly linked to the results where two individual genomes contain significant TE bursts. Is the Australian isolate one of these genomes? More clearly signposting this in the results (or even in Fig1?) will improve clarity as to why TE bursts are more likely to be observed in some isolates.*

**RESPONSE**: RIP mechanism is gradually weaker as described in Feurtey et al, 2022 (bioRxiv), yet dim2 is adding mutations and loses functionality (Möller et al, 2021). Even though it is not clear if loss of dim2 is enough to lose RIP functionality, we added the data of Möller et al, 2021 to Figure 1A. Still, we think that even if RIP is not active anymore, TEs and regions with RIP signature will remain in the genome for a long time.

*I have struggled with the ancestral reconstruction analysis and phylogenetic trees in the context of RIP. It was not clear to me from the methods if the AR was conducted on “de-RIPed” alignments? In my experience de-RIPing or removing sites in an alignment with RIP-like mutations increases pairwise sequence identity of alignments dramatically. I have concerns that the clades with high RIP mutations in figures 6-7 are grouping mainly on AT richness (text figure 5?), would trees constructed with de-ripped sequences give a better representation of the relationships within this TE family? I can see clearly for Deimos that the burst clade has very low RIP. This is less obvious for Cassini because the bursts are much smaller and a little nested within the tree. I did not understand the dot plots in all parts of Figure 6-7 and the statistical tests associated with these. More clarity around this analysis may address this question I have about ancestor reconstruction and the link to RIP-like mutations but these figures are not discussed in the current text.*

**RESPONSE**: We did not use de-RIP, as we were concerned by the accuracy of re-RIPping widely different TE families. We adjusted the text to avoid conflating branch lengths with age.

*All figures: Please consider reducing the number of panels. What are your main messages, can you remove “GC fragment” or “Large RIP affected Regions” etc etc? Do these different categories show very different things? If yes, then this should be more clearly discussed in the text for each figure, where this data remains.*

**RESPONSE**: We adapted the figures and removed panels showing the “RIP-index”.

*Figure 1: Parts A and B These are re-purposed figures from previous work, do you need to re-present both here, or is part B sufficient to show genome size is correlated with transposon copy number? Is the date of sampling important for isolates with TE bursts? Or perhaps indicating which isolates have a functional dim2 gene and therefore are capable of RIP?*

**RESPONSE**: Yes, Fig 1A-B are showing data from Badet et al, 2020. We added this information more prominently in the legend. We improved the graphics though to convey a clearer message about the geographic extent of the sampled genomic diversity.

*Fig1D: Allele frequencies? Allele counts? Frequency is between 0-1? May consider moving text “insertions were considered orthologous…”to main results text rather than the legend*

**RESPONSE**: We adjusted the figure

*Figure 2: Please consider moving the Legend or making it more visible. Currently it is embedded in the middle of part C and not clear that it is the color legend for other parts of the figure too.*

**RESPONSE**: We adjusted the figure

*Figure 4D, Figure 5: are all these panels needed?*

**RESPONSE:** We agree that the information is dense. We believe though that presenting the various aspects in conjunction is helpful to see links and limitations in our analyses. We improved the wording describing these figures.

*Figures 6-7: What is part D “GC content of the copy”?*

**RESPONSE**: We changed the text in the legend

*Could the authors please provide the fasta files used to construct phylogenetic trees? In their public datasets on github (if not too large) or Zenodo database?*

**RESPONSE**: We added these files on Zonodo (https://zenodo.org/record/7344421) as Supplementary Data.

***Part III – Minor Issues****: Editorial and Data Presentation Modifications*

*Please use this section for editorial suggestions as well as relatively minor modifications of existing data that would enhance clarity.*

*Reviewer #1: INTRODUCTION*

**RESPONSE**: We thank the reviewer and included all these helpful corrections mentioned below.

*Line 54: Please remove “and chromatin”, redundant with “histone modifications” in lines 53-54.*

*Line 110: Better to add “(Mycosphaerella graminicola)” after “Zymoseptoria tritici”.*

*Line 119: Please explain “MgDNMT” briefly (e.g. a DNA methyltransferase).*

*Line 131: “Escape of” → “Escape from”*

*RESULTS*

*Line 151: “5kb” → “5 kb”*

*Line 159: Please mention which TE category in Figure 2 (“MITE”, “Copia/Gypsy”, “LINE” or “other”) corresponds to “RLC” and “RLG”.*

*Lines 212-213: The percentage “20.7%” is not “more than half”.*

*Lines 236: The adverb “Similarly” is confusing since the traits mentioned in this sentence (LRAR and closest gene of burst copies) seem opposite between RII_Cassini and RLC_Deimos.*

*DISCUSSION*

*Line 335: “our a” → “our” or “a”*

*METHODS*

*Line 362: Delete “(Supplementary Figure S4)”.*

*Lines 396: “TheRIPper” → “The RIPper”*

*Line 405: Insert “(Supplementary Figure S4)” instead of it deleted from line 362.*

*REFERENCES*

*Lines 488-489: Please add the journal and volume.*

*Line 547: zymoseptoria → Zymoseptoria*

*Lines 582-584: The paper is now published (doi: 10.1093/g3journal/jkab068). Please cite the peer-reviewed one.*

*Lines 601-603: Please add the journal and volume.*

*Lines 654 and 655: Please cite the URL.*

*FIGURES*

*Figure 1D: Why is the font size of “9” on the x-axis larger?*

*Figure 2A: Please describe what the x-axis represents.*

*Figure 2B legend: Please add “(LRAR)” after “large RIP affected regions”.*

*###*

*Figure 2F: Could the statistical significance be shown? (e.g. Put an asterisk to the correlation coefficients supported with p < 0.05)*

**RESPONSE**: we adjusted the figure panel accordingly.

*Figure 5: Please explain “Mutations per bp” in the legend briefly. Is the mutation rate based on the consensus sequence for each family?*

**RESPONSE**: we thank the reviewer and added these corrections. The mutation rate is corrected for the length of the analyzed fragment and the number of detected copies.

*Supplementary Figures*

*Figure S3 title: “DTX_MITE_Gobblin” → “DTX_MITE_Goblin”*

**RESPONSE**: corrected

*Reviewer #2: The authors should point better to the panels in each figure to help the reader to follow what they claim*

**RESPONSE**: We improved the results text and re-ordered panels where helpful.

*Reviewer #3: Line 35: Is this only purifying selection or could there be other insertion preferences etc?*

**RESPONSE**: we changed the abstract and added references to the introduction concerning insertion site preferences

*Line 43: a genomic environments or genomic environment.*

**RESPONSE**: changed

*Line 49: define abbreviation (TEs)*

**RESPONSE**: we added the definition

*Line 51: "the potential of deleterious insertions into coding and regulatory regions", confusing wording, consider rephrasing.*

**RESPONSE**: we rephrased this sentence

*Line 59: "in all copies". RIP can target all copies, but does not necessarily hit every copy due to its stochastic nature.*

**RESPONSE**: We agree that this formulation was not clear and changed the text

*Line 85: explain positive effect.*

**RESPONSE**: we rephrased this sentence

*Line 96: RIP will also create long branches*

**RESPONSE**: we agree and rephrased this sentence

*Line 111: co-evolved in what way? Is it an obligate pathogen in wheat? Is it specific to cultivated varieties?*

**RESPONSE**: sentence clarified

*Line 113: comma after reference*

**RESPONSE**: changed

*Line 141: In Supplementary table 1 there are several isolates with a lower TE-content than 18.1%.*

**RESPONSE**: we corrected the TE content in the text

*Figure 1 seems to reproduce some information from the Badet et al., 2020 paper, such as the map. Is this necessary?*

**RESPONSE**: We agree Figure 1A and 1B are re-purposed from Badet et al, 2020. We made this even more clear in legend and main text, and improved the figure.

*Figure 1A: Difficult to see differences in TE content in figure 1A, scale only goes between 17-24 (very small). Australia has no estimated TE-content, or is it the most?*

**RESPONSE**: we agree that these values were not clear. We changed Figure 1

*Figure 1B: Figure text on x-axis is tiny.*

**RESPONSE**: We increased the font size

*Figure: 1C: What about non-MITE DNA transposons, are they all “other”? Both figure and text make it unclear what the abundance of these elements are. With this number of MITEs there must be some master copies, which are hijacked by the MITEs. They are likely <20 copies.*

**RESPONSE**: We added more information at the beginning of the results. Indeed, the “other” category contains full length DNA transposons, next to Helitrons, SINEs and non-autonomous retrotransposons. Showing all superfamilies by color would be more informative, but would also take away the focus from the more important points. The data is provided in the supplementary files.

*Figure 1C: “Between the same set of orthologous genes”, what if the genes are on the border of a large TE-island? Is this comparable to small insertion sites?*

**RESPONSE**: We adjusted the text for the relevant figure panel.

*Figure 1 C, a lot of windows are 100% TE. Presumably this is due to nesting. Does that affect the results?*

**RESPONSE**: Was this question possibly intended for Figure 2C? There is significant nesting and many TEs are fragmented. Yet we think by only extracting the coding regions for the phylogenetic reconstruction allows us avoid confounding effects due to fragmented copies. We tried to visualize this in Supplementary Figure S4. To make our point clearer, we also added more information to Supplementary Figure S4 and more information to the methods.

*Table S2 contains a massive amount of information. This is good in terms of reproducibility and open science. However it makes it difficult to find the information pointed to as in text references. e.g. Line 145. Perhaps a more readable table with a summary of important information, like what superfamilies given families are in, is needed.*

**RESPONSE**: We now improved the figure representations at the level of TE classifications (e.g. Fig. 3, results text). We hope that this will allow for a clearer overview. Hence, referring to Table S2 becomes only necessary to reproduce our analyses. See also below.

*Line 145-146: Neither figure 1C nor supplementary figure 1 are showing good comparisons of the number of families belonging to superfamilies in the different genomes.*

**RESPONSE**: We added the number of copies to Supplementary Figure S1. The information is also available in Supplementary Table S2 including a reference in the main text.

*Line 151: why 5 kb? Does “centered around” include the TE itself, or only the flanks? If the former then many of the retrotransposons may be larger than 10 kb and would have skewed values due to this.*

**RESPONSE**: We chose 5kb as the distance between genes is around 1-2kb, and we wanted scan TE presence in larger windows. These windows do not contain the TE itself. We clarified the text for both points.

*Line 156 and 159: Is the GC association only due to RIP?*

**RESPONSE**: Fig 2F (now Fig 3D) shows a strong negative correlation between GC content and RIP, and while RIP probably has a strong impact on the GC content, there are other potential mechanisms influencing the GC content). We adjusted the text.

*Supplementary figure 1: All text is way to small*

**RESPONSE**: We agree, yet, as this is a supplementary figure at high resolution, we think that the digital version should be zoomable unless printed out. We find it difficult to convey the full data intended to be represented otherwise. More succinct visualizations are available as main figures.

*Figure 2A: Label X axis. Y axis is TE density? The colours should be stated here as well, this is an issue for many of the figures. I recommend either clearly having the legend in part A, or prominently at the side in each figure.*

**RESPONSE**: Thank you. We adjusted the figures accordingly.

*Figure 2B: The fact that there are more TEs in core likely reflects that there is more DNA in the core. Should show as a proportion. Same with telomere and LRAR.*

**RESPONSE**: We added plots showing the TE density (number of TEs per bp) to compare.

*I question whether setting a cutoff for low GC at < 50% is meaningful when the genomes contain 51% - 52% GC.*

**RESPONSE**: a cutoff of 50% separates the regions clearly into regions with high vs low gene density. We believe that even though cutoff is somewhat arbitrary that the interpretations are not affected.

*Figure 2 C: What are the weird peaks around 43% and 49%?*

**RESPONSE**: GC content was rounded to 1% creating this uneveneness in the display.

*Line 168: "Longer TE copies tend to be located in already TE rich niches", is that length fragment? Throughout the text and figure 2 it is not clear what “fragment” refers to.*

**RESPONSE**: We rephrased this sentence to make it clear that we refer to the full length of the consensus sequence

*Supplementary figure 3A: are all clades bursts? The black bar on the outside is not that informative. The axes should be labeled clearer, not evident what's meant by copy and sequence.*

**RESPONSE**: we changed Supplementary figure 3 according to the main figures 6 and 7

*Line 175: “We find that high-copy TE families tend to also have more variable copy numbers among 19 genomes, indicating ongoing activity of individual families”. Why does this indicate ongoing activity? Also, this is more of a discussion point.*

**RESPONSE**: we rephrased this sentence

*Line 179: “with the shorter copies belonging to the non-autonomous MITEs” And what is the longest copy?*

**RESPONSE**: we added this information

*Line 182: RIP is not clock-like, so it cannot be considered “age” for ripped sequences.*

**RESPONSE**: We agree. We changed “age” to “genetic distance” and reformulated the text where we referred to Kimura distances.

*Figure 3: I think the figure has labeling issues. It looks like the fourth column is DTX_MITE_Goblin, which has a consensus length of 226 bp, but the bar plot seems to show that it's over 5000 bp. Likewise the fifth column should be RLC_Deimos at 6356 bp, but the plot shows a very small size, currently unreadable. I didn't go through every column, but this should be checked.*

*Figure 3C: log scale would be better to show lengths as many look like 0. Perhaps for nucleotide diversity as well.*

*Figure 3F: The text in the plot is too small. It is also difficult to tell what TE Superfamilies the different data points correspond to (except for MITE).*

*Figure 3: There is no panel G or H? These sound like the same as supplementary figure S2A and S2B*

**RESPONSE**: we thank the reviewer. Indeed, there were multiple labeling issues in this figure. We corrected the labeling and added a legend for the superfamily color.

*Line 198: 'the factors'*

**RESPONSE**: changed

*Figure 4 B: I don't understand this plot at all*

**RESPONSE**: We modified the figure by removing the background and added more information to the figure, legend and results. This figure panel should show the direction of change in sequence characteristics from reconstructed parent to a newly created copy. We modified the text describing this figure.

*Line 200: What superfamilies do these belong to? It is quite difficult trying to figure out what type of elements these are.*

**RESPONSE**: we added this information

*Figure 5: The colours do not show up on many of the violin plots. Another way of labeling would help.*

**RESPONSE**: we have now colored the outline, and added NA for missing values

*Figure 5: Colors in legend and figure text do not match. Legend: “Red not part of burst”. Text: “copies in burst (red)”*

**RESPONSE**: changed

*Figure 5: Confusing to have families that have both burst/non-burst and families that only have non-burst(?) in the same figure.*

**RESPONSE**: RLG_Luna and RLG_Sol do not show indications of a recent burst. We agree this makes this figure more challenging to read and, therefore, added “NA” for the burst.

*Figure 6: It looks like there may be a lot of bursts of intermediate age, does this affect the results? What do the dot plots show? It’s not clear what the x axis refers to.*

**RESPONSE**: (Now Fig. 7) We improved the description of the figure in the main text and legend. The dotplot refers to the changes in RIP-like mutations from the estimated ancestral state to the offspring. It is true that the tree may show a series of older bursts creating less copies than the most recent burst. However, analyzing older bursts carries more uncertainty in terms of resolving factors related to the burst initiation.

*Figure 6 - 7: Do trees have support?*

**RESPONSE**: support values for the trees are now available in the new Supplementary Data S3 (on Zenodo)

*Line 260: 'of TEs'*

**RESPONSE**: changed

*Line 384: While Kimura distances can be calculated on RIPped sequences, they mean something very different from how Kimura distance is normally discussed in TE literature i.e. these are not mutating at constant rates and don’t refer to age.*

**RESPONSE**: We changed “age” to “genetic distance” and reformulated the text where necessary.

*Reviewer #4: Minor comments:*

*Lines 78-79: Preferred insertion site of TEs, is it worth mentioning here that many of these are “TA” sequences, which is also linked with RIP?*

**RESPONSE**: We could not find any indication of AT rich preferred insertion sites and a link to RIP in the literature. This is an interesting thought though and we prefer mention this in the discussion now.

*Lines 151-152: is TE “insertion” misleading? You analysed 5kb windows around each annotated TE? Or you only analysed TE’s where you clearly had an empty site in another genome? (possible as very few TEs fixed)*

**RESPONSE**: We now refer more clearly to “TE copy” where appropriate throughout the manuscript. We agree that “insertion” indicates an active process, which was not intended. We think the use of “copy” is a more neutral term, and only kept “insertions” when we were talking about the active process

*Line 173-174: RLC and RLG not defined, Focus of figures 1 and 2 are mainly of these families and MITEs, might be worth clarifying early in results why you focus on these families and define them.*

**RESPONSE**: we added the missing definitions, keeping in mind the RLG is in the process of being redefined.
