# Supplementary material for: Recent transposable element bursts are associated with the proximity to genes in a fungal plant pathogen
Source: PLoS Pathog. 2023 Feb 14;19(2):e1011130. doi: 10.1371/journal.ppat.1011130 (PMC9970103; doi:10.1371/journal.ppat.1011130)
Supplement: Attachment — Submitted filename: Response_reviewers.docx [file ppat.1011130.s010.docx]

Dear Dr Thomma,

Thank you very much for the excellent peer-review process. We are glad to hear that our revisions were well received. We are now submitting a revised manuscript addressing all the minor issues raised in the second round.

With best regards,

Daniel Croll

*######################################*

*Reviewer's Responses to Questions*

***Part I - Summary*** *Please use this section to discuss strengths/weaknesses of study, novelty/significance, general execution and scholarship.*

*Reviewer #1: The manuscript has been improved after the first review process. The discussion becomes deeper, which attracts interest in the relationship of TE bursts with the insertion into specific gene niches and the activity of a genomic defense mechanism (RIP). The finding of the variation of possible burst mechanisms among TE families would be also interesting. The paper can provide a subset of arranged information for TE science in plant pathogens. I suggest modifying some minor issues listed follows.*

*Reviewer #3: The revised version of the manuscript is a significant improvement on the original. The authors have done an excellent job addressing all of the reviewers comments. The clarity has increased dramatically, making for a pleasurable read. I think the manuscript is more or less ready for final publication in its current form, though I have noted some minor errors and have a couple suggestions listed below. However there is one point that I highly recommend the authors look over closely. In Figure 6 D it appears the burst copies of RLC_Deimos are mostly inserted into genes. I would double check these to make sure that the TE ORFs aren't miscalled as genes. If it's not an error this fact could be worth highlighting more in the text.*

**Response**: We thank the reviewer for this important point. We checked our gene annotation for the subset of the reference genome IPO323, with RLC_Deimos copies. Ultimately, only a small number of the genes has a function described that could be associated with the well described RLC_Deimos functions. Most of the annotated genes have a function clearly independent from Copia coding regions, and the ones that are not assigned a known function are unlikely Copia elements as well. Overall, we are convinced that only a very small percentage of all genes with a RLC_Deimos insertion are indeed TEs wrongly assigned as genes. The above information is added to the relevant results section.

*Additionally, I am aware of the debate regarding the family name "gypsy". I highly recommend using Ty3 as the name for the family, or at the very least Ty3/mdg4, especially as these are fungal TEs. In any case please check the consistency as sometimes RLG is used and sometimes mdg4 through out the manuscript.*

**Response**: We agree with the naming conventions and changed mdg4 to Ty3 in the introduction, and additionally kept the Wicker code abbreviations for the rest of the text and figures 1-4. We also added "Ty1/Copia", to retain consistency with the introduction. Additionally, the TE legend of Figure 3 was accidentally removed and is now added for completeness

*Reviewer #4: (No Response)*

***Part II – Major Issues: Key Experiments Required for Acceptance*** *Please use this section to detail the key new experiments or modifications of existing experiments that should be absolutely required to validate study conclusions.

Generally, there should be no more than 3 such required experiments or major modifications for a "Major Revision" recommendation. If more than 3 experiments are necessary to validate the study conclusions, then you are encouraged to recommend "Reject".*

*Reviewer #1: The major issues pointed at the first review process seems to be solved and I did not find additional major issues.*

*Reviewer #3: (No Response)*

*Reviewer #4: (No Response)*

***Part III – Minor Issues: Editorial and Data Presentation Modifications*** *Please use this section for editorial suggestions as well as relatively minor modifications of existing data that would enhance clarity.*

*Reviewer #1: Line 142: Delete "The loss of".*

**Response**: changed

*Line 211: RLC_Deimos seems an extreme case of "The GC content of high copy TE families" "lower than 50%" rather than "the exception".*

**Response**: we agree this should be highlighted and adjusted the text

*Line 225: There is no "Figure 4H".*

**Response**: thank you for pointing this out. This was in fact supposed to be a reference to Figure 4F
 *Line 246: The percentage of "a large part of TE copies" that "remain in large RIP-affected regions" is missing.*

**Response**: adjusted. The stated number mistakenly combined both TEs that remained in LRAR and jumped into LRAR (now line 248f)

Lines 268-270: The description about RIP seems redundant within the sentence.

**Response**: We rephrased the relevant sentence.

*Lines 275-276: RII_Cassini seems to show a "similar pattern of temporal escape
from RIP facilitating a burst" like RLC_Deimos though with a smaller degree.*

**Response**: We agree in principle, we are not convinced though that we should this somewhat speculative point.

*Line 663: Correct "xx".*

**Response**: changed
 *Line 709: 67-U139 → 67-77*

**Response**: changed

*line 711: Bmc → BMC*

**Response**: changed

*Fig 1B: Why Copia/mdg4 instead of RLC/RLG?*

**Response**: We adjusted the naming for RLC/RLG in text and figures, and only kept the more complete explanations including the older names for completeness in the introduction.
 *Fig 7E, 8E and S3E: The values of the scale seems inconsistent between the pylogenetic tree and the correration plot. If the scale for the correlation plot indicates log10 value, please mention that.*

**Response**: We thank the reviewers for pointing this out. We adjusted this in the relevant plots.

*Reviewer #3: Line 120: "high number of low highly similar" -> confusing language, consider rephrasing.*

**Response**: rephrased
 *Line 137 : "Has both produced both" -> remove one both; missing an "and"*

**Response**: changed (now Line 138-139)

*Line 189: "is" -> are*

**Response**: We think that the original sentence was correct
 *Figure 1: details of map construction should be in methods*

**Response**: we moved the information from the legend to the methods. We also removed the link to openmaps, as we now used the map provided in ggmap in R. We also moved the version and source information to R

*Figure 2: the LRAR density pattern is surprising, is it labelled properly?*

**Response**: We re-checked the data and think this is correct.
 *Line 197: "LINES" no s*

**Response**: changed
 *Figure 4: Much improved, great work!

Line 303: "General" -> generally*

**Response**: changed (now line 304)
 *Line 316: "play also" -> also play*

**Response**: changed (now line 317)
 *Line 402: One reason why copies near genes may not be targeted is due to RIP spill over, where adjacent sequence is mutated along with the targeted duplicate region. This could be mentioned here to explain the "secure niche".*

**Response**: We do not fully agree with this statement as the lack of finding such patterns could also be the result of strong counterselection rather than a somehow "secure niche". We believe that the current text is sufficient in terms of laying out possible explanations.
 *Supplemental figure 5: "Netherland" -> Dutch; error with "accessory chromosome" labels.*

**Response**: adjusted the country label. We re-checked the "accessory chromosome" labels and find these to be correct.

*Supplemental table 1: are the underscores in the "isolate" column intentional?*

**Response**: These were intentional. Some isolates have different names, yet we agree this is not adding any information, and we removed underscores for clarity

*Reviewer #4: This is a substantially revised manuscript that addresses most of the issues raised in my previous review.

One outstanding area that I feel could be explained more clearly is the ancestral reconstruction dotplots shown in figures 7 and 8. I still struggle to fully understand this analysis and feel that some additional explanatory text in the results here may help clarify, or in the methods lines 523-529. For example the phrase “We imported the trees into R using read.tree..” Which tree did you import as the starting point for this analysis the highest scoring ML tree? OR “We added metadata using ddplyr…” Which I assume means you assigned each sequence in your tree (each tip) the data associated with that particular TE copy. Would the authors like to state more clearly what the significance of a strong correlation or lack there of means? (i.e. strong correlation indicates not a lot of change through time? Or the inverse?)*

**Response**: We thank the reviewers for pointing out that the methods were not yet clear enough and complemented the relevant method text (now lines 526 ff) as suggested.

*Other minor typos spotted throughout the text:
Line 71: tough ◊ though*

**Response**: changed
 *Line 142: “the loss of cytosine methylation was lost..”*

**Response**: corrected

*Line 160: “Escape from genomic defenses including silencing is a likely the major driver of TE dynamics.” This sentence stuck out to me because silencing was not really measured in this study.*

**Response**: We agree and improved the sentence.

*Lines 174-176: “Half (n=106) … half to retrotransposons (n-59)”. Does not seem quite correct to use “half”.*

**Response**: We thank the reviewer for pointing this out. We rewrote the sentence to make clear that around half the TE copies were either DNA or retrotransposons.

*Line 189-190: “We found no overall association between TE copies and large RIP-affected regions.” I think this comes with a small caveat that LRAR can completely destroy some TE copies making them impossible to detect.*

**Response**: This is indeed a possible scenario over longer evolutionary time spans. However, given that RIP stops adding mutations at ~20-30% divergence, we think that over the short intra-specific divergences studied here, TEs should not vanish entirely in this context.

*Line 324: insert “by” …were strongly affected BY RIP prior to the bursts.*

**Response**: changed
